# The association between short video addiction and perceived writing competence among Chinese EFL learners: the mediating role of decreased attention control and learning burnout

**DOI:** 10.3389/fpsyt.2026.1761069

**Published:** 2026-02-26

**Authors:** Yanning Chen, Chuang Xu, Jimin Hu, Jian-Hong Ye

**Affiliations:** 1 School of Languages and Cultures, Hunan Institute of Technology, Hengyang, Hunan, China; 2 School of Marxism, Hunan Institute of Technology, Hengyang, Hunan, China; 3 Department of Foreign Languages, Ganzhou Teachers College, Ganzhou, Jiangxi, China; 4 Faculty of Education, Beijing Normal University, Beijing, China

**Keywords:** decreased attention control, English-learning burnout, perceived writing competence, short video addiction, stimulus-organism-response

## Abstract

The cognitive patterns of the teenagers have been reshaped by the digital era through pieces of information and instant responses. According to the Goldilocks hypothesis, there might be a non-linear relationship between the use of digital media and learning outcomes. This study mainly focuses on the part of excessive usage, which is the addiction to short videos. The risks it brings, such as distraction and poor working memory, may pose a unique threat to the learning tasks that require a high level of cognitive engagement. Although there is an increasing number of scholars have investigated the relationship between short video addiction and students’ learning performance, few studies focusing on the relationship between these two variables in the context of English as a foreign language. Therefore, the present study seeks to propose a model for predicting how students’ short video addiction is related to their English writing task. Participation in the study was 559 Chinese university students. The results of structural equation modeling revealed that short video addiction was significantly negatively correlated with students perceived writing competence; among them, both decreased attention control and English-learning burnout played the mediating roles in this relationship. These findings reveal that addiction to short videos beyond the “optimal range” is associated with the depletion of cognitive and emotional resources and is linked to lower perceived English-writing competence, providing important insights for educators to enhance students’ writing competence by intervening in attention control and learning burnout.

## 1 Introduction

English writing is a high-level, multi-dimensional and recursive cognitive activity. As a core skill of a foreign language, it is a multi-level interaction from pre-writing to the writing and revision stages (1). This is not merely a presentation of knowledge; it also involves a more profound cognitive process of knowledge transformation (2). Although this cognitive process is crucial for the development of writing, so far, research on the linkage between addiction to the short-form videos and learning mainly concentrates on its impact on macro academic achievements (such as overall performance), while neglecting its impact on specific, higher-level skills, such as English writing. Given the wide popularity of applications like TikTok, whose downloads are 875.67 million globally in 2024, this gap is particularly important (3). These platforms are deeply integrated into students’ daily lives, and they provide not only video content but also social and interactive functions (4). According to Goldilocks hypothesis, the association between digital media usage and academic performance is not a simple linear negative correlation. Their relationship may present an inverted U-shaped curve. That is, moderate and purposeful use may bring benefits such as providing information and promoting collaboration, while insufficient or excessive use may be associated with adverse consequences (5). This hypothesis shifts academic discussions from whether to use to how to use it reasonably. However, in the current digital environment, the design of short video platforms is aimed at maximizing user stickiness, which is prone to excessive using unconsciously, making users more likely to be at the lower end of the inverted U-shaped curve. It is reported that about 21.6% of Chinese students have the symptoms of addiction to short videos (6). Xie et al. (7) stressed its importance as a necessary but harmful substance for students’ everyday routines. As Brunborg and Andreas (8) emphasized the overuse of social media can bring negative effects, highlighting the urgency to examine its specific impact on cognitively demanding tasks such as EFL writing.

To further understand those influences, it is necessary to analyze it within the broader context of how the digital age has reshaped the cognitive patterns of young people, who now face risks related to addiction of watching short videos such as attention fragmentation, decreased working memory capacity and weakened metacognitive regulation (7, 9). These changes are the new challenges for EFL writing, which requires the writer’s long concentration and logical thinking ability, but all of these are vulnerable to the “short, flat, and fast” stimulation modes of short video watching. Since EFL writing acts as an important link for cross-cultural communication and global literacy, it is necessary to understand in what way addiction deteriorates writing via cognitive and affective paths.

Based on this, Ye et al. (10) suggested that the harmful effects of short video addiction on young people have not been well studied, and further investigations are needed. Among the theories accounting for such dynamics, the SOR theory is particularly useful for explaining how addiction develops and what its outcomes are. This approach proposes that environmental stimuli (S) impact an organism’s internal state (O), which subsequently influences its behavioral responses (R), and it can serve as a valid paradigm to examine the associative effects between environmental cues and behavioral responses (11). Accordingly, the aim of this study is to theoretically reveal the pathways and mechanisms by which EFL writing competence is related with addiction to short videos. Therefore, we address the following two research questions:

RQ1: Does addiction to the short videos negatively relate to the Chinese EFL learners’ writing competence?

RQ2: Do the attention control and learning burnout mediate the relationship between addiction to the short videos and writing competence among Chinese EFL learners?

## 2 Literature review and hypotheses

### 2.1 The stimulus-organism-response framework

The SOR framework once used in environmental psychology, highlights how external stimuli impact an individual’s internal psychological state and thus provoke behavioral reactions (11). The SOR model has been used in a variety of contexts, such as online environments and social platform ecommerce (12). Although a large number of studies have demonstrated that the SOR model can predict addictive behaviors, it is mainly applied in the research of short video purchases and user behaviors (13). In this study, we have adopted the term “addiction” since the aim of this study is to investigate the behavioral and psychological factors behind the overuse of the short-form video platform. Short video addiction (SVA) means an individual’s inability to control the duration and frequency of using short-video apps, ultimately causing damage to their physical and mental health (8). The SOR framework can provide the theoretical support for explaining how SVA ultimately associates with writing competence through the mediating mechanisms of the emotional pathway (burnout) and the cognitive pathway (attention).

### 2.2 Research hypotheses

#### 2.2.1 The direct relationships

Previous studies have made some contributions to the negative effects of short video addiction on students’ learning (14–18). They have widely related it to self-efficacy of English academic ability (16), academic achievement (14), learning well-being (15), silent classroom behavior (17) and academic engagement (18). However, empirical evidence regarding the impact of SVA on learning tasks that require concentration (e.g., English writing) is still insufficient. The SVA is negatively related to students’ learning, but it is not clear whether it has the same negative connection with tasks that require long-term concentration, such as English writing. Therefore, to understand whether there is a negative correlation between SVA and students’ English writing by examining their writing competence. Perceived English-writing competence (PWC) refers to a person’s subjective confidence in their ability to complete assigned English writing tasks within rhetorical and cultural contexts. It covers a series of creative activities from conception, drafting, revision, to finalization (19). We put forward the following hypothesis:

H1: Short video addiction is negatively related to perceived English-writing competence.

Although watching short videos is mainly for entertainment, immoderate use may harm users’ cognitive functions. Empirical research indicates that overuse of short-form video platforms is significantly associated with weakened individual self-control, and self-control is precisely the key psychological basis for maintaining attention (4). More importantly, neuroscience research has further confirmed that frequent social media use can induce adaptive changes in the brain’s cognitive functions and structures, affecting core cognitive abilities including attention control (13). These mechanisms have also been echoed in public discussions, with many reports criticizing the distracting and addictive nature of social media (20). Besides, some scholars also confirmed that excessive usage of social media can result in behavioral addiction, and prolonged usage correlations with decreased attention control. (4, 21). Decreased attention control (DAC) indicates the decline in an individual’s ability to regulate and allocate attention resources after long-term excessive exposure to short videos (4). Therefore, we put forward the following hypothesis:

H2: Short video addiction is positively related to decreased attention control.

Academic burnout is students’ feelings of exhaustion brought on by high study demands, their cynical and distancing attitude toward their university work and their sense of inadequacy or lack of accomplishment as students (22). Huang (16) has proved that short video addiction can significantly impact academic burnout. The mechanism by which short video addiction predicts academic burnout is also applicable to the specific English learning fields, which can effectively fill the research gap in this direction. English-learning burnout (ELB) refers to a decrease in learning enthusiasm and a sense of success due to the recurring sensation of tiredness that causes, as well as a decrease in learning ability (16). The existing action paths have significant applicability in the context of English learning. Being addicted to short videos will occupy the time continuity and cognitive resources needed for English learning. Especially, it will be detrimental to language acquisition processes that require frequent exposure, such as vocabulary memorization and reading input. This will indicate learning procrastination, reduced efficiency, and intensify the sense of powerlessness and fatigue in English learning. (14, 23). In addition, the problems such as anxiety, low sense of achievement and emotional exhaustion induced by addiction to short videos may further externalize as “foreign language learning anxiety” in English learning, specifically manifested as resistance to classroom interaction and negative expectations of English grades. All these highly overlap with the behavioral and emotional dimensions of English learning burnout (24). Thus, we put forward the following hypothesis:

H3: Short video addiction is positively related to English-learning burnout.

Piolat et al. (25) emphasize that writing is an accumulative outcome that requires continuous thinking and attention to generate. Classic writing cognitive models (such as 26, 27) indicate that writing involves the coordination of multiple sub-processes such as planning, translation, and revision, and these processes compete for the individual’s limited cognitive resources. However, higher levels of SVA predict lower levels of attention control (21). Additionally, Furthermore, the immediate satisfaction brought by short videos has a significant negative correlation with an individual’s tolerance for mundane tasks. This makes the writers instinctively avoid activities that require concentration, such as brainstorming and revision work, etc. This habit could undermine the cognitive foundations required for writing (13). And become the risk factors for users’ perceived English-writing competence. Therefore, we put forward the following hypothesis:

H4: Decreased attention control is negatively related to perceived English-writing competence.

An increasing number of scholars are finding that burnout is a key predictor of university students’ lower learning performance (28–31). Feeling exhausted from studying, being cynical and uninterested in one’s academics, and feeling unworthy as a student were all considered signs of learning burnout (28). English-learning burnout means that the feeling of repeated fatigue in the process of learning English, which related to a decrease in learning interest, achievement, and learning ability. However, up to now, there are no conclusive results. Each research shows differences in the strength and direction of this relationship. Although scholars have highlighted that burnout significantly negatively linked with learning performance (28–30), Fiorilli et al. (32) find that there is no influence between burnout and learning performance. Besides, in Atalayin et al. (31) ‘s study, learning burnout even positive correlation with learning performance. To fill this gap, it is necessary to verify this relationship again. Therefore, we propose the following hypothesis:

H5: English-learning burnout is negatively related to perceived English-writing competence.

#### 2.2.2 The mediate relationships

In this study, we suggest that the DAC constitutes the internal mediating pathway from the external stimulus of SVA to the behavioral response of poor perceived writing competence according to the SOR theoretical framework (11). A prior study has also confirmed that short video addiction contributed to users’ decreased attention control (4), which is the organism in this study. Specifically, the complex cognitive task of English writing requires individuals to engage in continuous and in-depth information processing and to delay gratification (such as repeated revision and conception) (52). However, an individual with decreased attention control ability will find it difficult to effectively allocate and maintain their cognitive resources, which is manifested as: being easily distracted during the writing process, having difficulty entering and maintaining a “flow” state of deep thinking, and having their working memory occupied by irrelevant information fragments (33). Ultimately, these internal cognitive dilemmas suggest a negative link between DAC and English writing, such as disordered paper structure, insufficient depth of content, and frequent language errors. Therefore, we put forward the following hypothesis:

H6: Decreased attention control mediates the relationship between short video addiction and perceived English-writing competence.

SVA is an important antecedent that accounts for learning burnout. Addictive behaviors will encroach upon the continuous cognitive input required for learning, thereby triggering learning procrastination and inefficiency, and intensifying students’ sense of powerlessness (7). Furthermore, the immediate and high-intensity entertainment experience provided by short videos can negatively related to students’ interest in learning (15). The decline interest in learning has been proven to be the core factor of learning burnout (16). Secondly, although no concluded results in the previous studies, there is still a large amount of evidence shows that the states of physical and mental exhaustion, decline in learning interest and lack of sense of achievement manifested by learning burnout can successfully predict students’ poor academic performance and achievement (28–30). When it comes to the complex task of English writing, which requires sustained concentration and emotional investment, exhausted students are more likely to exhibit behaviors such as difficulty in conceiving ideas, procrastination in writing and low willingness to revise. Therefore, we propose that SVA is an effective stimulus to the ELB (organism) and indirectly linked to poor perceived writing competence. So, we propose the following hypotheses:

H7: English-learning burnout mediates the relationship between short video addiction and perceived English-writing competence.

### 2.3 Research framework

**Figure 1:**
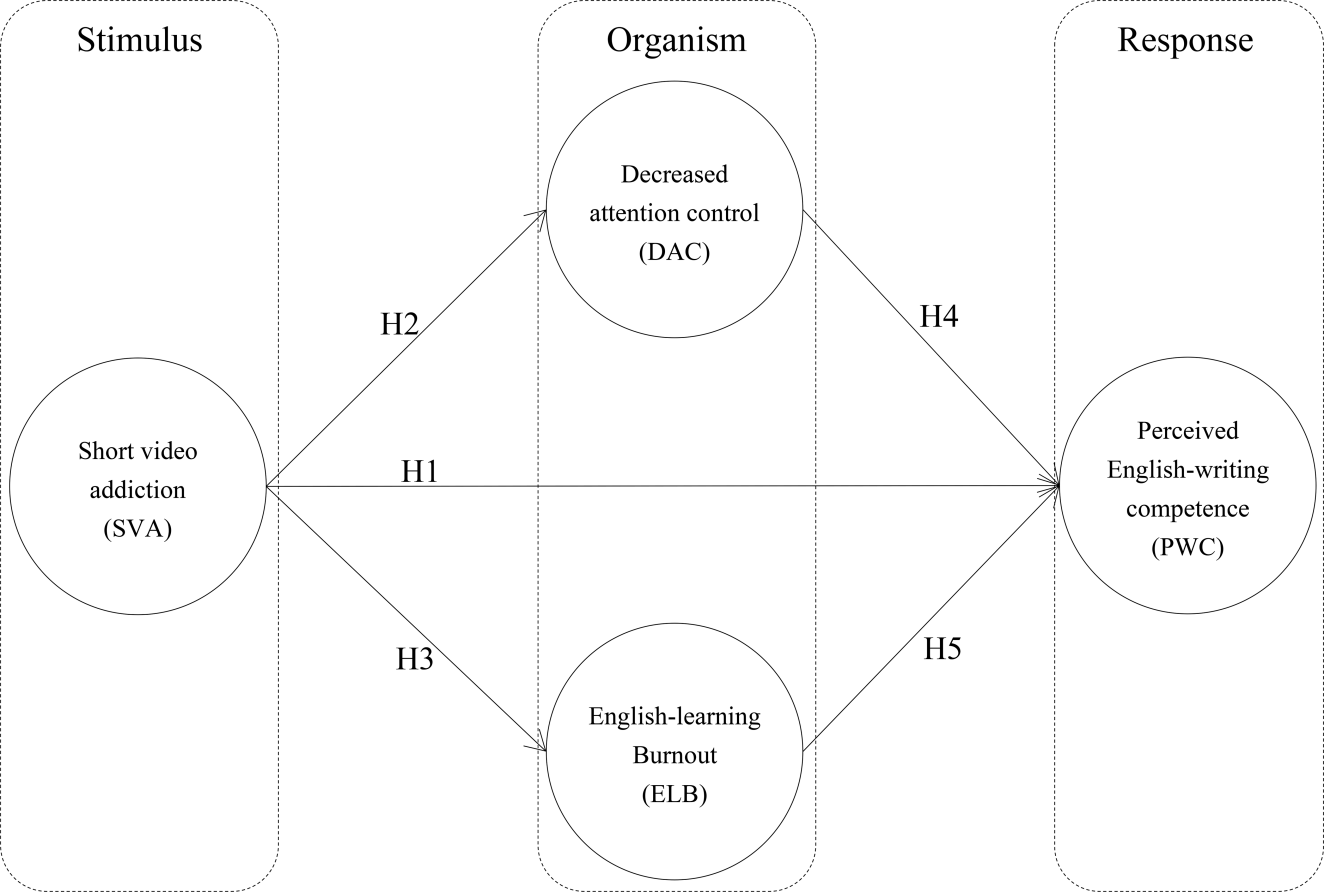
SVA framework.

In order to fill the gap of previous research, we propose the following framework in **Figure 1** SVA framework.

## 3 Methodology

### 3.1 Procedure

The Academic Committee of the Hunan Institute of Technology gave this study approval. The Wenjuanxing platform was adopted to conduct an online survey to gather the data. We used snowball sampling in August 2025 and gave the survey anonymously to Chinese full-time university students who had been using short-form video apps for a long time. The selection of this group is based on the premise that students with more extensive short video usage experience can more effectively reflect the typical consumption behaviors of this population. To lower the risk of homogeneity in the initial sample, stratified sampling was used for the initial “seed” participants based on academic years, disciplinary background (the humanities, natural science, or military), and frequency of using short videos. In order to prevent snowballing based solely on homogeneous social circles and to guarantee enough variability within the sample to effectively reveal the hypothesized path relationships, each participant was required to recommend peers with differentiated backgrounds (such as different majors, gender, and household registration type).

The online questionnaire began with a statement of informed consent that described the purpose, background, research objectives, data use, and procedure of the study. It also stated that the privacy and anonymity of the participants would be maintained and gave contact details of the principal investigator of the study. On the informed basis, participants could click an option that they had read and agreed to all the contents of the consent form. “I have understood the above content and agree to participate in this study”, to continue with the formal survey. Besides, “Have you completed at least one semester of English courses?” is the screening question. To ensure the accuracy of the survey subjects. Only the students who select “Yes” can answer all the whole questions based on their actual situation. After the quality checks and invalid questionnaires were excluded, 500 valid responses were left; the response rate was 89.44%. Descriptive analysis, reliability analysis, validity analysis, common method bias test, and correlation analysis on SVA, DAC, ELB, and PWC were conducted by using SPSS 27. To test the effect of SVA on students’ PWC, and to test the mediating roles of DAC and ELB, a structural equation model (SEM) was developed in smart PLS 3.3.9.

### 3.2 Participants

The participants selected for this study were Chinese students who regularly use short-form video apps and have completed at least one semester of English coursework. Detailed information regarding the participants’ gender, academic year, subject category, household registration type, as well as their average number of days using short-form video apps per week and average daily time spent watching short videos, is provided in **Table 1**.

**Table 1:** Descriptive information.

Variables	Details	Percentage
Gender	Male:243	48.6%
	Female:257	51.4%
Academic years	Freshmen:143	28.6%
	Sophomore:123	24.6%
	Junior:114	22.8%
	Seniors:120	24%
Subject category	The Humanities:216	43.2%
	Natural Science:218	43.6%
	Military:66	13.2%
Household registration type	Rural Citizen:236	47.2%
	Urban Citizen:264	52.8%
Average days of using short-form videos platform per week	1-3:90	18%
	4-6:148	29.6%
	Every day:262	52.4%
The average daily time spent watching short videos	1–2 h:121	24.2%
	2–3 h:158	31.6%
	3–4 h:106	21.2%
	>4 h:115	23%

### 3.3 Measurements

This study adopts a questionnaire to collect the data. The measurement items in the questionnaire were derived from past literature. Some of the measures have been adapted to suit the SVA environment, and the questionnaire was reviewed by three professors. Besides, a Likert five-point scale was used to score the design components, with responses ranging from strongly disagree (1) to strongly agree (5) (34). Considering that the data collection for this study was carried out in China, the questionnaire design adopted a bilingual version in Chinese and English. To ensure the accuracy of translation, the study introduced the back translation method, which was verified by Werner and Campbell (35) and can effectively guarantee that the content of the questions in the target language version is completely consistent with that of the original questionnaire. During the specific implementation process, the research team entrusted a professor and a PHD student majoring in related fields who is proficient in both Chinese and English to conduct a detailed translation of each item of the questionnaire.

#### 3.3.1 Short video addiction

This study selected the SVA measurement items developed by Ye et al. (15). This scale measures the participants’ awareness of addictive characteristics during the use of short videos through 10 specific items. Typical measurement items include: “Stopping watching short-form videos makes me feel down.” The scale score is positively correlated with the severity of addiction. The higher the score, the more serious the tendency toward addiction. After examination, the Cronbach α was 0.91, the AVE was 0.64, the CR was 0.93, and the FL was (0.730-0.826), all meeting the recommended standards by Hair et al. (36).

#### 3.3.2 Decreased attention control

This study employed Ye et al. (4)’s measurement items for DAC. This tool consists of six measurement items and is specifically designed to assess participants’ self-control ability of attention. Typical measurement items include: “After using short-video apps, I often have difficulty concentrating when doing other things.” The scale score is positively correlated with the degree of decline in attention control. The higher the score, the more severe the impairment of attention regulation ability. After reliability and validity tests, the Cronbach’s αwas 0.89, the AVE was 0.65, the CR was 0.92, and the FL was (0.785-0.823), all of which met the recommended standards (36).

#### 3.3.3 English-learning burnout

This study selected the ELB measurement items developed by Huang (16). The original measurement was fully considered and precisely adapted to the specific learning scenarios where university students experience burnout during their English learning process. This measurement specifically includes 10 items, such as “I performed poorly in the process of learning English and even had the thought of giving up.” The higher the score, the more severe of their learning burnout. After reliability and validity tests, the Cronbach’s α was 0.89, the AVE was 0.60, the CR was 0.91, and the FL was (0.733-0.813), all of which met the recommended standards (36).

#### 3.3.4 Perceived English-writing competence

This study adopted 8 items for PWC from Akhtar et al. (19), specifically including “I understand how to write sentences using correct tenses; I think sentence structure is not a challenge for me in writing.” After reliability and validity tests, the Cronbach’s α was 0.89, the CR was 0.92, and the AVE was 0.57, FL was (0.727-0.799), all of which met the recommended standards (36).

## 4 Results

### 4.1 Preliminary analyses

When evaluating convergent validity, we use two indicators, Factor Loading (FL for short) and Average Variance Extracted (AVE for short), for measurement. Both the FL and AVE must be greater than 0.50. In addition, **Table 2** demonstrated that the discriminant validity was good since the square roots of the AVE were higher than the correlation coefficient between the variables that were related to them. Then, this study further examined the validity by using the Heterogeneity-Singularity Trait Ratio (HTMT), which is the ratio of the correlation between different traits and the correlation within the same trait. The results of **Table 3** shows that the HTMT values between each of the two constructs was less than 0.85, indicating that each construct had good discriminant validity (37), and **Table 4** showed the Pearson correlation (36).

**Table 2:** Discriminant validity.

Variables	DAC	ELB	PWC	SVA
DAC	0.806			
ELB	0.241	0.775		
PWC	-0.235	-0.309	0.757	
SVA	0.294	0.340	-0.191	0.800

The diagonal line’s value is the square root of AVE.

**Table 3:** HTMT criterion.

Variables	DAC	ELB	PWC	SVA
DAC				
ELB	0.267			
PWC	0.261	0.341		
SVA	0.323	0.373	0.203	

**Table 4:** Pearson correlation.

Variables	M	SD	SVA	PWC	ELB	DAC
SVA	3.159	0.884	1			
PWC	3.077	0.902	-.161**	1		
ELB	3.228	0.86	.296**	-.303**	1	
DAC	3.31	0.954	.286**	-.232**	.257**	1

**p < 0.01, *p < 0.05.

### 4.2 Goodness of fit assessment

SRMR is the standardized variance between the actual and predicted correlations, which is a comprehensive measure of fit for the data (38). In PLS-SEM, SRMR is a key indicator to measure the quality of the overall model setting (39). When its value is less than 0.08, it can be considered that the correlation difference between the model and the data is within an acceptable range, thereby indicating that the model has a basic overall fit degree (40). The analysis confirms that the model for this research gives a good fit of the data with an SRMR value of 0.064. As shown in **Table 5**.

**Table 5:** Model fit.

Fit Indices	Saturated model	Estimated model
SRMR	0.057	0.064
Chi-Square	1331.133	1335.714
NFI	0.835	0.835

### 4.3 Structural equation model

**Figure 2:**
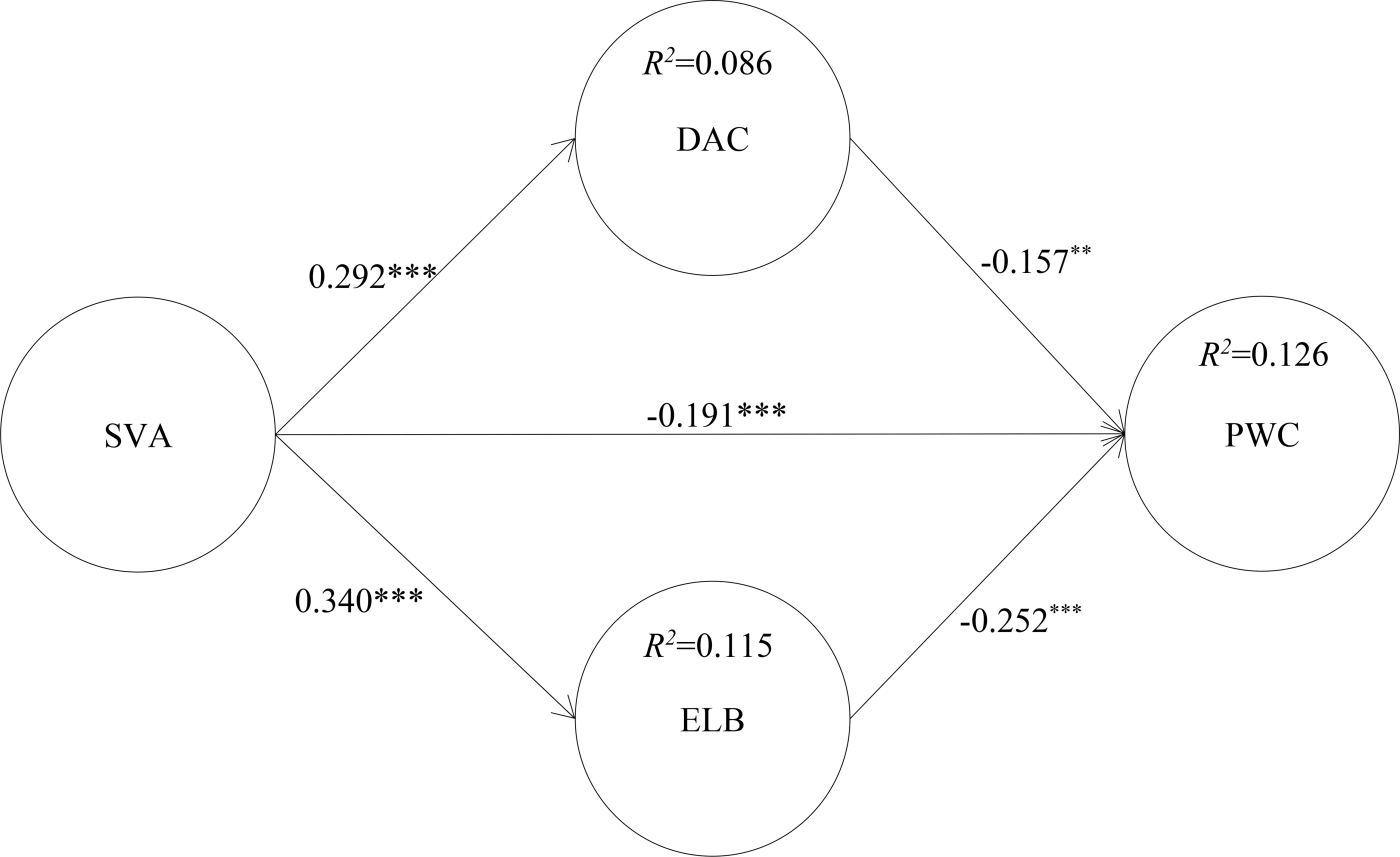
Structural model. ***p < 0.001, **p < 0.01, *p < 0.05.

After the reliability and validity tests, we used path coefficients and P-value analysis to test the structural model using Smart PLS. The test results are shown in **Figure 2**. SVA was positively related to PWC (β=-0.191, p < 0.001), thus H1 was supported. Besides, SVA was positively related to DAC (β=0.292, p < 0.001) and ELB (β=0.340, p < 0.001), thereby H2 and H3 were supported. Additionally, DAC (β=-0.157, p < 0.01) and ELB (β=-0.252, p < 0.001) negatively influenced PWC, supporting H4 and H5. For H6, the path coefficients of SVA -> DAC -> PWC (β = -0.046, t-value = 2.643, p < 0.01; LL = -0.084, UL = -0.017) and for H7 the path coefficients of SVA -> ELB -> PWC (β = -0.085, t-value = 4.245, p < 0.001; LL = -0.132, UL = -0.050) were significant, thus supporting hypotheses H6 and H7.

## 5 Discussion

Results show that the daily short video viewing time of the participants is concentrated between 1 and 4 hours, among which 24.2% spend 1 to 2 hours, 31.6% spend 2 to 3 hours, 21.2% spend 3 to 4 hours, and 23% spend more than 4 hours. This data distribution is highly consistent with the research of Brunborg and Andreas (8) whose report indicates that teenagers use social media for about 2.5 hours a day on average, and nearly one-third of them use it for nearly 3 hours. These two results corroborated each other in the main intervals, enhancing the reliability of this study. Under the SOR framework, this study agrees with Ye et al. (17) and gathered multi-dimensional information such as academic year, subject category, and household registration type, making the data of university students more comprehensive. The path analysis confirmed that all nine hypotheses put forward in the research were supported by the data gathered from 500 effective participants.

### 5.1 Direct relationships

The result confirmed that SVA correlates with PWC negatively, which is in line with the Goldilocks hypothesis that excessive use of digital media predicts negative consequences. It theoretically breaks through the limitation of existing research that mostly focuses on generalizing academic elements (e.g., happiness and engagement) (14, 16, 17), and provides evidence for fragmented cognition eroding deep writing ability, revealing the inherent conflict between addictive behaviors and higher-order cognitive tasks. First, short videos are thought to only take up a limited amount of time. People unconsciously devote a lot of time to watching short videos, which fills the time that should be used for everyday work activities and causes task procrastination (41). People are addicted to watching short videos because such content can bring instant satisfaction (42). Simultaneously, writing term papers is frustrating, boring, and stressful for EFL learners (43). Consequently, if students who watch short videos excessively indulge in short videos, they may delay completing unpleasant academic tasks (44). This conclusion provides a precise target for educational intervention in practice, warning educators to go beyond the simple time limit and focus on reshaping students’ concentration and deep-thinking habits.

Besides, the result also shows that SVA is positively correlated with DAC, that is, addicted users are easily to be distracted and have concentration problems. This conclusion is in line with Ye et al. (4)’s study that the more severe the addiction to short videos is, the lower the level of attention will be. Liu et al. (21) also found that addicted people have significant attention problems when watching short videos, and their attention is difficult to maintain. As a result, the changes in brain activity patterns and the deterioration of users’ attention control are positively correlated with the severity of SVA. This is consistent with the research of Tian et al. (13), who recommended that long-term viewing of short videos can relate to habitual distraction, making it difficult to remain focused on low intensity, stimulating tasks such as writing tasks.

Finally, the result also confirmed that SVA has a significant positive link with ELB, align with Huang’s research (2025). This explains that excessive viewing of short videos can consume users’ cognitive resources, add to their psychological burden during the learning process, and trigger a tendency towards avoidance. This result resonates with the mobile phone addiction research, suggesting that the SVA issue should be paid attention to in education to alleviate students’ learning burnout (14, 23). Besides, this study finds that ELB significantly negatively related to PWC, which concludes the previous studies and supports both Fard et al. (30) and Kljajic et al. (53).

### 5.2 Mediate relationships

This study confirmed the mediating role of DAC, which is consistent with the (SOR) theoretical framework (11). This mechanism reveals the path by which stimuli ultimately trigger specific behavioral responses by altering the intrinsic cognitive state of the organism. Addicted students have more attention problems and produce low-quality term writing assignments. Therefore, when people encounter external stimuli such as social media, short videos, and message notifications, they need to work hard to regulate their unstable behavior and stay focused on their goals (45). In addition, those with attention control problems have difficulty resisting temptation and completing tasks (44), and they tend to take longer than average to retain their desires and devote themselves to attention work. (46, 51). Thus, in complex cognitive tasks, students with decreased attention control ability have great difficulties in English writing (33).

Besides, ELB is also an effective mediator. This result supports the findings of the study by Salmela-Aro et al. (47), which found that academic burnout makes students have less interest and desire in learning activities, eventually relating to their adverse learning outcomes (48). In particular, students with greater addiction to short-form videos had the higher level of ELB, which would subsequently associate to their poor perceived writing competence. Addictive behaviors not only consume students’ study time, but also undermine the emotional foundations needed for writing. In addition, for EFL learners, English writing itself has very high requirement and be regarded as a complex and difficult task. Then, the students need to maintain their attention so as not to stray from the topic during the writing process (49, 50). Learning burnout makes it difficult for students to maintain the willingness and endurance to conceive and revise in English writing, which needs continuous concentration and emotional investment, and ultimately manifests as a decline in writing quality, which is align with previous research (28, 29).

### 5.3 Implications

Research and practical applications will be greatly influenced by these results. Firstly, the findings of this study have enriched the Goldilocks hypothesis in the field of digital media usage. The results have confirmed the underlying logic mechanism behind the overuse of digital media. Specifically, addiction to short videos not only relates to the depletion of cognitive control resources, manifested as decreased attention control, but also becomes a risk factor for learning burnout, ultimately linked to students’ poor perceived writing competence. This conclusion is no longer limited to a simple description of the correlation between excessive use and adverse outcomes but provides empirical support for explaining why excessive use predicts negative effects and through what specific pathways it generates the association. Furthermore, the research also suggests that the “optimal range” of digital media usage mentioned in the Goldilocks hypothesis should vary depending on the nature of the learning task. Tasks that require deep thinking and sustained attention, such as EFL writing, are more sensitive to the bad consequences of the excessive use of digital media. Therefore, the threshold for the “optimal range” of digital media usage for such tasks is likely to be lower than that for receptive learning tasks like reading. This provides a direction for future research, which is to analyze the Goldilocks hypothesis of digital media usage by considering the specific types of learning tasks. Finally, this study concludes the mixed findings of burnout on learning performance and fills the research gap in the linkage between learning burnout and the perceived academic performance of English learners, especially in English writing task, which also enriches the relevant literature on short video addiction.

This study also provides practical ideas for related educational practices: In the digital age, educational work does not mean completely prohibiting students from using digital technologies. Instead, it is necessary to guide students to find the optimal range of digital media usage that suits their own circumstances and meets the requirements of specific learning tasks, and to maintain this. Meanwhile, it is essential to notice the students’ digital media usage status, and early identification should be made of those students who have already shown tendencies of addiction to short videos and are about to exceed the usage risk threshold. Timely targeted intervention and guidance should be carried out to prevent them from falling into learning difficulties due to excessive use.

### 5.4 Limitations and further studies

There are some limitations in this study. First, in the process of questionnaire collection, this study failed to adequately consider control variables (such as English proficiency, overall academic performance, or study time allocation). This omission may lead to deviations in the estimation of the relationships between variables. To conduct a more rigorous test of the relationship between excessive use of short videos and English learning, future research suggests including some control variables, such as basic English proficiency and academic performance, and adopting a longitudinal design or experimental intervention to establish a temporal sequence. Besides, in the future, other contextual factors such as the university atmosphere, teacher and student relationships, and family environment, which have not been considered, can also be added as moderators. These variables may be also related to the relationship between SVA and PWC. Incorporating these variables will help improve the ecological validity.

Second, the sample employed the snowball sampling method. To enhance the general applicability to all university students, future research could adopt the probability sampling method to further verify the universality of this model. In addition, considering that the attention control ability of teenagers is still developing, they may be more susceptible to the addiction to short videos. Future research should include participants of different age groups to clarify exactly how the influences of SVA on people has developed step by step.

Third, the measurement tool of English-writing performance used in this study mainly rely on participants’ self-reports rather than the objective evaluations. This approach may more reflect the subjective evaluations of participants regarding their own writing skills. Therefore, in future research, in order to more accurately and comprehensively assess English writing performance, objective writing tasks can be included. For example, actual writing tasks can be designed, and researchers or evaluators can conduct the assessment directly based on clear and unified scoring criteria (such as language accuracy, text coherence, content completeness, etc.).

Finally, the cross-sectional data revealed significant correlations among the variables but failed to confirm the causal relationship between addiction and poor writing competence. Although the data support the mediating path in the model, the interpretation of the temporal sequence and causal mechanism between the variables requires great caution. Future research could adopt longitudinal tracking plans or experimental intervention methods to verify the causal chain and dynamic process of this model more rigorously.

## 6 Conclusion

The results show that: (1) SVA is positively correlated with the DAC and ELB; (2) Both DAC and ELB are negatively correlated with PWC; (3) DAC and ELB fully mediate the relationship between SVA and PWC. These findings suggest that short video addiction has a significant negative relationship with perceived writing competence indirectly. Its impact on perceived writing competence is through the students’ decreased attention control and English-learning burnout. Conversely, addiction to short videos can directly link to a decline in attention control ability and burnout in learning English. Therefore, teachers and parents should try to reduce the students’ English-learning burnout and encourage students’ self-regulation skills development. Because university students are the main hope and force for future social development, their pursuits in academic field have a profound impact on their personal development and the long-term development of society. This research therefore has a dual theoretical and practical significance.

## Data Availability

The raw data supporting the conclusions of this article will be made available by the authors, without undue reservation.
